# Climate Change Increases Drought Stress of Juniper Trees in the Mountains of Central Asia

**DOI:** 10.1371/journal.pone.0153888

**Published:** 2016-04-21

**Authors:** Andrea Seim, Gulzar Omurova, Erlan Azisov, Kanaat Musuraliev, Kumar Aliev, Timur Tulyaganov, Lyutsian Nikolyai, Evgeniy Botman, Gerd Helle, Isabel Dorado Liñan, Sandra Jivcov, Hans W. Linderholm

**Affiliations:** 1 Regional Climate Group, Department of Earth Science, University of Gothenburg, Gothenburg, Sweden; 2 Central-Asian Institute for Applied Geosciences (CAIAG), Bishkek, Kyrgyzstan; 3 Kyrgyz National Agrarian University (KNAU), Bishkek, Kyrgyzstan; 4 Innovation Center of Phytotechnologies, National Academy of Sciences of the Republic of Kyrgyzstan, Bishkek, Kyrgyzstan; 5 University of Central Asia, Bishkek, Kyrgyzstan; 6 Republican Scientific Production Centre for Decorative Gardening and Forestry, (RSPCDGF), Tashkent, Uzbekistan; 7 German Centre for Geosciences, Climate Dynamics and Landscape Evolution, Helmholtz Centre Potsdam, Potsdam, Germany; 8 Forest Research Centre, Instituto Nacional de Investigación y Tecnología Agraria y Alimentaria (INIA-CIFOR), Madrid, Spain; 9 Institute of Geology and Mineralogy, University of Cologne, Cologne, Germany; Chinese Academy of Sciences, CHINA

## Abstract

Assessments of climate change impacts on forests and their vitality are essential for semi-arid environments such as Central Asia, where the mountain regions belong to the globally important biodiversity hotspots. Alterations in species distribution or drought-induced tree mortality might not only result in a loss of biodiversity but also in a loss of other ecosystem services. Here, we evaluate spatial trends and patterns of the growth-climate relationship in a tree-ring network comprising 33 juniper sites from the northern Pamir-Alay and Tien Shan mountain ranges in eastern Uzbekistan and across Kyrgyzstan for the common period 1935–2011. Junipers growing at lower elevations are sensitive to summer drought, which has increased in intensity during the studied period. At higher elevations, juniper growth, previously favored by warm summer temperatures, has in the recent few decades become negatively affected by increasing summer aridity. Moreover, response shifts are observed during all seasons. Rising temperatures and alterations in precipitation patterns during the past eight decades can account for the observed increase in drought stress of junipers at all altitudes. The implications of our findings are vital for the application of adequate long-term measures of ecosystem conservation, but also for paleo-climatic approaches and coupled climate-vegetation model simulations for Central Asia.

## Introduction

Uzbekistan (UZ) and Kyrgyzstan (KG) belong to a region commonly referred to as Central Asia. This largely semi-arid to arid mountainous region is particularly vulnerable to ongoing and predicted climate change [1, 2]. Temperatures increased during 1901–2009 [3–5] and are predicted to further rise during winter, while heat waves will become more frequent during summer [1]. The high diversity in topography and land uses modify the spatial and temporal distribution of precipitation, and rainfall amounts are expected to further increase in winter, especially in eastern UZ, but decline in summer across the entire region [1, 6].

Observed impacts of anthropogenic climate change include an increase in health risks [6], decrease in agricultural crop productivity [3], increase in desertification [7], increase in floods and advanced glacier recession [8, 9], and alterations in phenological phases of species and habitat shifts [10–12]. Particularly, a potential increase in drought-induced tree mortality and decline in forest vitality [13–16] and changes in forest productivity [17] would have dramatic consequences for the forest ecosystem in this region. Besides potential threats to the environment, socio-economy and human health, the unequally distributed water resources in this region are likely to further increase political tensions between the countries [18, 19].

The mountains of Central Asia are classified as one of the 34 biodiversity hotspots in the world [20]. Under climate change scenarios (A2 and B2) mountain coniferous forest ecosystems are likely to be among the most affected [21, 22]. Juniper (*Juniperus* spp.) dominates with around 80% of the forested areas at mid-to high elevations in Central Asia [23]. For UZ, Botman [21] estimated a future upslope shift of juniper forests of 350–400 meters, consequently leading to an overall spatial reduction of the juniper belts by 350 meters due to less favorable pedogenic conditions at the tree line.

Variations in annual tree growth that predominantly reflect weather conditions can be used to detect impacts caused by recent climate changes in response to an increased CO_2_ content in the atmosphere during the industrial era. In UZ, the mountain conifer forests are confined to the northern Pamir-Alay in the southeast and western Tien Shan ranges in the northwest of the country. Due to political unrest and restricted accessibility, only one dendroclimatological investigation has been conducted in the past decades [24]. In adjacent KG, research has been done on various economically important tree species in the Tien Shan and northern Pamir-Alay Mountains such as walnut (e.g. [25]), spruce (e.g. [26] and references therein; [27]), and juniper (e.g. [28–30]). For spruce, distinct and statistically significant trends in climate response as a function of elevation were detected, where spruce growth benefits from warm temperatures at high elevations, while it is limited by water supply at low altitudes [26]. However, the impact of climate on juniper has been shown to be less clear, e.g. [28, 29].

This study aimed to evaluate the climatic signal of juniper along altitudinal gradients in and across eight regions from eastern UZ to eastern KG. We investigated spatial trends and patterns in recent juniper growth using a new and extensive tree-ring width (TRW) network of 33 sites from the northern Pamir-Alay and Tien Shan mountain ranges in UZ and KG. We compared averages of climate, annual TRW increments, and tree growth-climate responses, as a function of elevation, between 1935–1964 and 1982–2011, and mapped differences across the entire study region. Results were discussed in the light of observed changes in climate, tree growth and climate sensitivity in Central Asia and similar regions.

## Material and Methods

### Geographical setting and sampling sites

**Fig 1:**
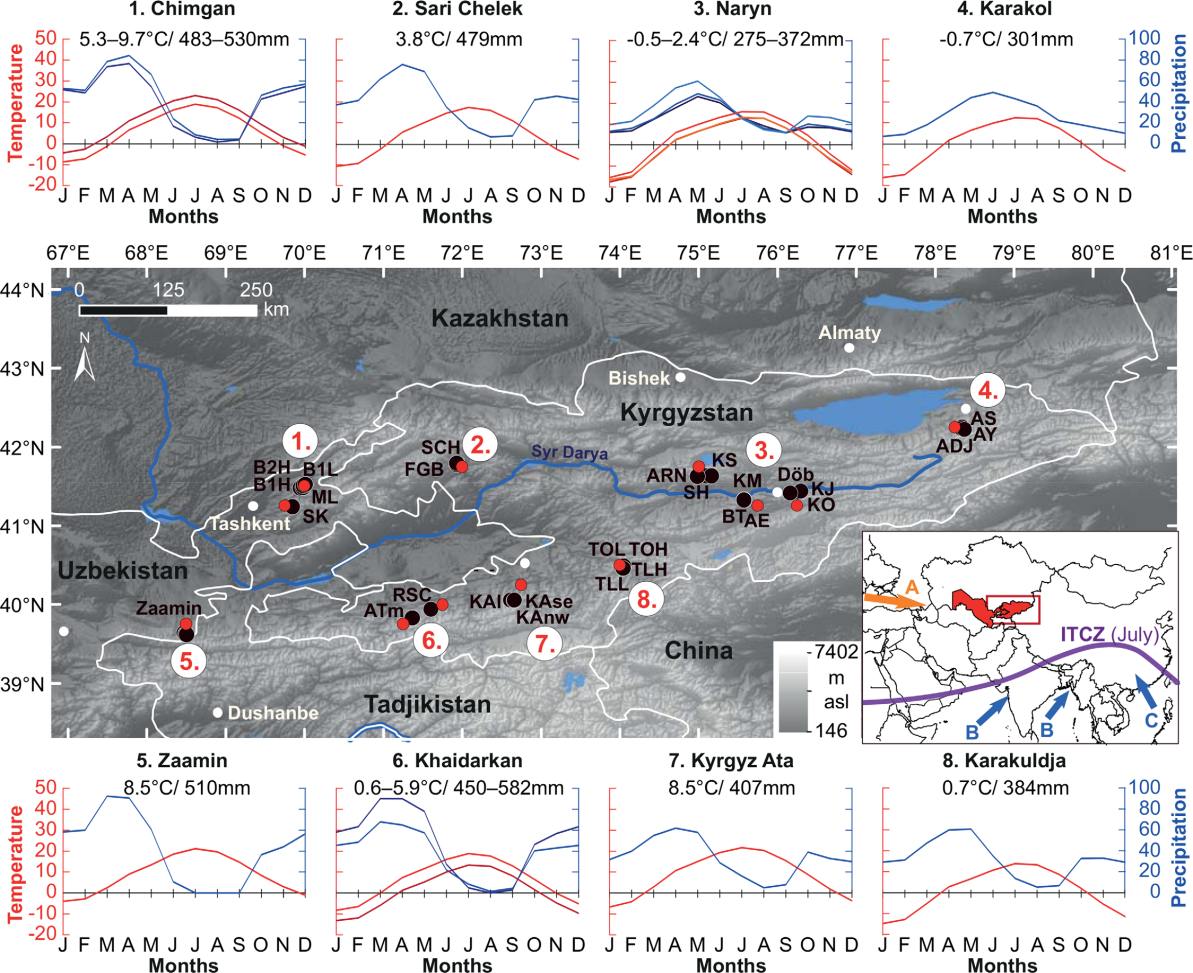
Juniper sampling sites (black dots) and closest CRU grid point data (red dots) in Uzbekistan and Kyrgyzstan. Westerly winds (A; orange arrow in inset) dominate the study area causing continental climatic conditions at the sites. Climate charts show absolute annual (numbers) and monthly temperature means (red shades) and precipitation sums (blue shades) for all CRU grid points for each region over the period 1961–1990. Monsoonal influences (blue arrows) are depicted for the Indian Summer Monsoon (B) and East Asian Summer Monsoon (C), respectively, while ITCZ stands for Intertropical Convergence Zone (purple line; after Lutgens and Tarbuck, 2001 [31]). Topographic features are indicated by digital elevation model data in grey colors.

During 2012 and 2013, 1069 juniper trees were sampled at 33 sites ranging from 1267 to 3020 m above sea level (asl) within the 68.49°–78.37°E and 39.63°–42.24°N domain (Fig 1, S1 Table). The sampling was conducted with permission from local forestry districts and National Parks in eight regions located in the northwestern (Zaamin, UZ), northern (Khaidarkan and Kyrgyz Ata, KG) Pamir-Alay, western (Chimgan, UZ; Sari Chelek, KG) and the central (Karakuldja, Naryn and Karakol, KG) Tien Shan mountain systems stretching from west (eastern UZ) to east (all of KG) across our study area (Fig 1). We selected juniper sites close to or at the local upper and lower tree lines (S1 Table) to get a better understanding of juniper climate responses along elevational gradients.

The climate in the study area is controlled by two pressure systems: during winter, a northeasterly inflow of cold air originates from the Siberian High, and in summer, the eastern extension of the Azores High causes a northwesterly inflow of, by then, dry air. Thus, the climate is extremely continental with cold winters, hot/dry summers, and a precipitation maximum in spring (see climate charts in Fig 1). A decreasing aridity trend from west to east during summer can be observed, altering the climate from semi-arid (until Naryn, central KG) to wet continental at the most eastern region in KG (Karakol; Fig 1). Moreover, at the highest elevation sites (> ~2500 m asl), the climate is strongly affected by altitude displaying a more alpine climate regime, especially in Khaidarkan, Karakuldja, Naryn and Karakol with low annual temperatures (annual temperature averages are given in the climate charts in Fig 1).

*Juniperus* (*J*.; locally called ‘Archa’) is the dominant taxa at mid- to high altitudes (1200–3500 m asl). Following altitudinal zones from low to high, three main subspecies, *J*. *seravschanica* Kom. (JUSE), *J*. *semiglobosa* Regel (JUSM), and *J*. *turkistanica* Kom. (JUTU), form open forests with trees up to 8–10 meters in height on shallow soils, i.e. lithic leptosols (FAO classification; [32]). While juniper forests are highly abundant in eastern UZ and western KG, they are successively replaced by spruce (*Picea* sp.) in central KG and eastwards. At its eastern distribution limit in KG, only fragmented patches of juniper occur, where the trees often develop prostate stem forms.

### Tree-rings and climate data

From all 1069 trees, a minimum of two cores per tree were extracted and 1882 samples were annually dated following standard dendrochronological procedures [33, 34], described in detail in Seim *et al* [35]. For the development of the individual site TRW chronologies, we first eliminated biases caused by temporally uneven sample replication by applying a power transformation [36] to each raw TRW series. Dimensionless indices were computed as residuals and age-induced growth trends were removed by applying flexible age-dependent splines [37] using the software ARSTAN [38]. This data-adaptive and, at the same time, deterministic standardization procedure uses splines that become progressively inflexible with increasing tree age [37]. The variance of each final site chronology was stabilized [39] based on the interseries correlation (Rbar) [40], and we used robust biweight means of the power transformed, variance stabilized, TRW indices, for which the pooled autoregressive properties of the sites were retained. We truncated each chronology at a replication of *n*(*i*) < 5 series. The Expressed Population Signal (EPS) [40] as a measure for the common signal strength of a chronology is also provided in S1 Table for the overall common AD 1935–2011 period. It should be noted that for Zaamin (UZ), three composite chronologies for the three subspecies were developed from eight sites (see details in Seim *et al* [35]), which were subjected to the above mentioned procedure and used in this study.

Although several meteorological stations exit with data extending back to the early 20^th^ century [41], a consistent network of long temperature and precipitation data close to our sampling sites is still lacking. Hence, we used high-quality interpolated data from the Climate Research Unit (CRU) TS3.22 [42] that fully covers the 1901–2012 period. Temperature and precipitation records were extracted from the nearest grid points to our sampling sites (Fig 1) and normalized over the baseline period 1961–1990.

### Data analyses

Based on the 1935–2011 period common to all TRW data, we compared climate, tree growth and the growth-climate relationship from the earliest, 1935–1964, to the most recent, 1982–2011 period. This was done to attempt to find a causal link between juniper growth and climate response to observed changes in climate during the 20^th^ century. For the climate data, anomalies of temperature and precipitation from each CRU grid point were used to produce means for the most contrasting seasons: for winter (previous year December to current year February, pDJF) and summer (June–August, JJA). Changes in juniper growth were calculated by averaging the TRW indices over the two 30-year periods. We used Pearson correlation statistics to investigate the growth-climate relationships for the full 1935–2011 and the two shorter (1935–1964, 1982–2011) sub-periods. This was done using monthly climate values, as well as the seasonal temperature means and precipitation sums. Additionally, both TRW chronologies and climate data were high-pass filtered using a 10 year spline to investigate the growth-climate relationship during summer in the high-frequency domain.

To examine spatial and temporal changes in winter and summer climate, annual tree growth, and climate-growth responses to summer temperature and precipitation, the period differences were entered as point data in ArcMAP 10.1 [43]. Areas with no data coverage were estimated by an inverse distance weighted (IDW) interpolation technique [44]. This method fills missing cell values using a distance-weighted average of neighboring points. The power parameter *p* defines the influence of the weights. We used twelve neighboring points and *p* = 2, to give higher weights to closer points and progressively decreasing weights to distant points.

The impact of elevation on the climate data, the so-called lapse rate, was neglected in the study since we used interpolated grid point data. However, changes in tree growth and climate sensitivity were analyzed as a function of altitude.

## Results

### Growth-climate response (1935–2011)

The growth-climate responses obtained for the full 1935–2011 period highlight the diversity in climate sensitivity of junipers across different elevations and regions (S1 Table). Only junipers in southwest KG showed a distinct precipitation response at lower elevations (Khaidarkan and Krygyz Ata until ~2500 m asl; Karakuldja until ~2000 m asl) and a temperature response at higher altitudes towards the end of the growing season (S1 Table). Juniper at all other sites and regions responded either to a lack in water supply or to high temperatures in spring, summer or during the full growing season, i.e. April to October (S1 and S2 Tables).

Moreover, only a few sites responded to one single climate parameter (S2 Table). TRW in KAnw (Kyrgyz Ata, KG), TOH (Karakuldja, KG) and ADJ (Karakol, KG) was positively correlated with temperature, while TRW in SK (Chimgan, UZ), TLL (Karakuldja, KG), SH1 and ARN (both Naryn, KG) showed distinct precipitation associations at monthly to seasonal scales (S2 Table). The remaining 26 sites exhibited either a mixed climate response or changing responses over the course of the year. Strong responses to drought, expressed as negative/positive correlations with temperature/precipitation at the same time, were observed for ZNP-JUSE (Zaamin, UZ) and most of the sites in Naryn, central KG, especially in summer but also during the entire vegetation period (S2 Table). Regarding changes in climate sensitivity, six (five) sites experienced changing temperature (precipitation) responses during all seasons (S2 Table).

**Fig 2:**
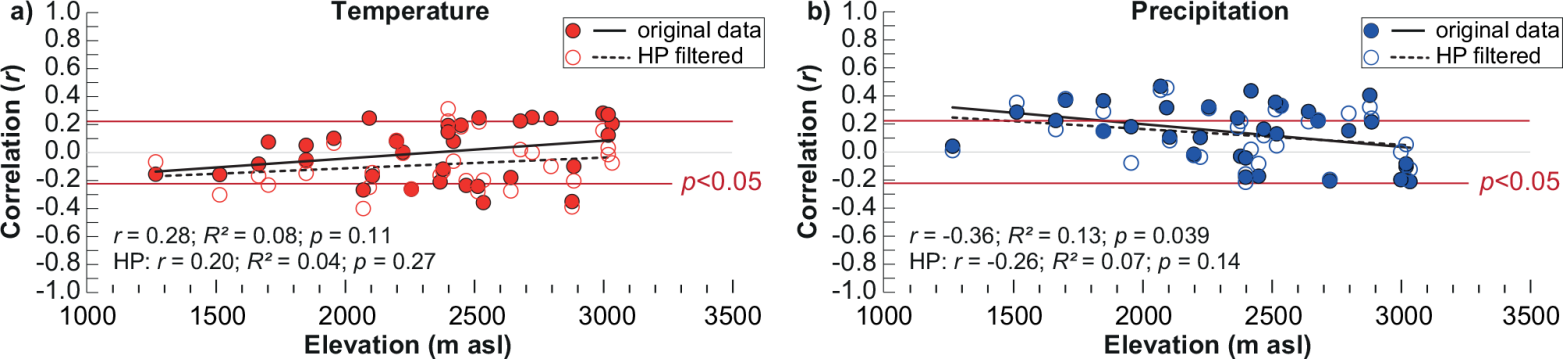
**Climate response for June–August a) temperature and b) precipitation of all 33 juniper sites as a function of elevation for the AD 1935–2011 period.** Correlation values are shown for the original and filtered (10-year high-pass (HP)) data.

In summer, a total of seven sites located at higher elevations showed significant positive summer temperature responses, while drought was the dominant growth limiting factor at lower altitudes (Fig 2; S2 Table). Thus, an overall linear increasing trend in temperature sensitivity and decreasing trend in precipitation sensitivity with increasing elevation, was observed for both original and high-pass filtered data (Fig 2).

### Changes in climate, juniper growth, and climate responses

**Fig 3:**
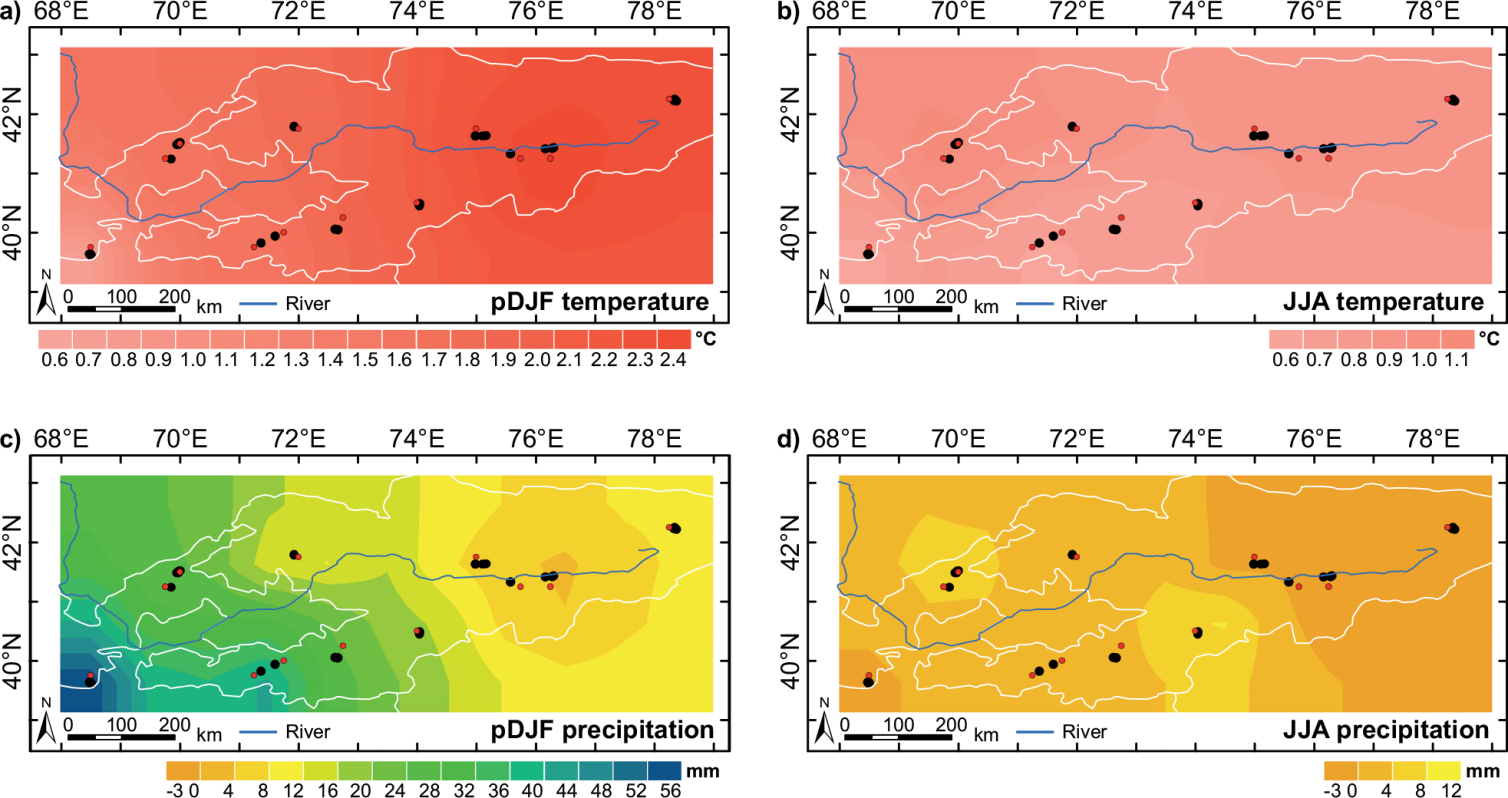
Changes in climate from the 1935–1964 to 1982–2011 period spatially interpolated from the climate data (CRU grid point, red dots) closest to the tree-ring sites (black dots) for a) winter (previous year December to current year February, pDJF) and b) summer (June–August; JJA) temperature and c) winter and d) summer precipitation.

From 1935–1964 to 1982–2011, winter temperatures increased by 0.6°C to 2.4°C from eastern UZ to eastern KG, whereas the highest increase in summer temperatures of 1.1°C was recorded in central and eastern KG (Fig 3A and 3B). Winter rainfall increased by 56 mm in eastern UZ but remained more or less constant in central and eastern KG (Fig 3C). The smallest changes in rainfall were observed during summer in the eastern parts of both countries, increasing by 12 mm in northeastern UZ (Chimgan) and southern KG (Karakuldja) (Fig 3D).

**Fig 4:**
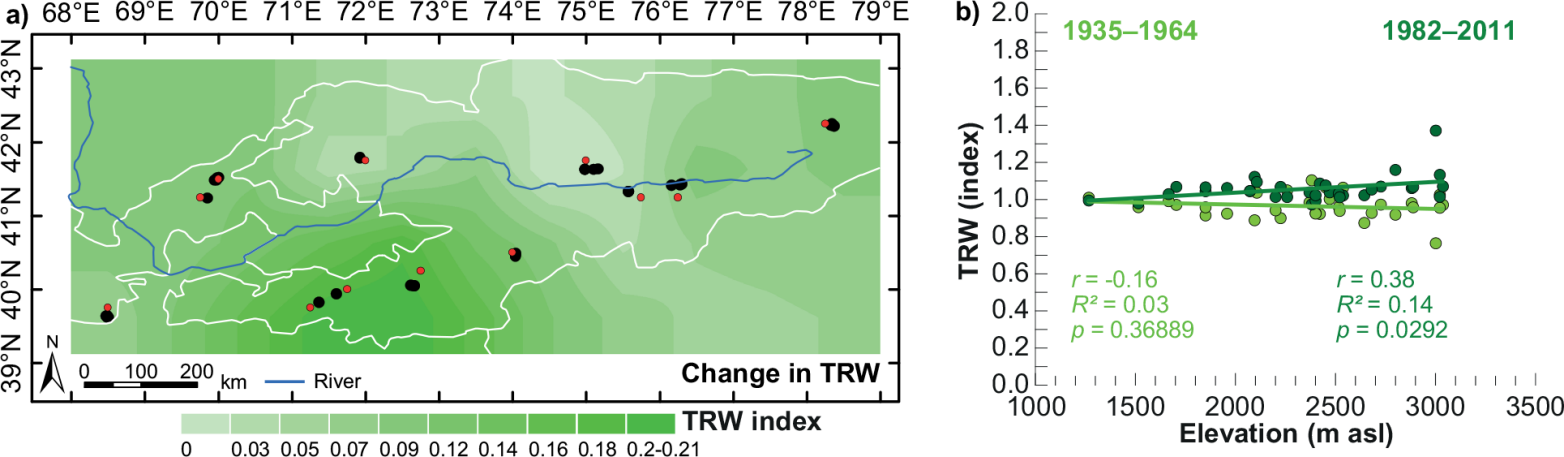
**Changes in juniper growth from the 1935–1964 to 1982–2011 period a) spatially interpolated from the 33 sites (black dots) and b) shown as a function of altitude.** Red dots denote the sites closest CRU grid point.

Changes in annual tree growth using detrended TRW indices indicated slight but significant (*p*<0.01) increases in increments by ~0.2 index units, being most pronounced mainly at the highest altitudes (> ~2500 m asl) in southwestern KG (Khaidarkan and Kyrgyz Ata) (Fig 4A and 4B). On the other hand, no changes or even slight decreases in annual growth were observed at the lower elevation sites in Zaamin (UZ) and at sites in Sari Chelek and Naryn (KG). However, considering the temporal variability among the individual TRW chronologies over the last decades (S1 Fig), those values might not be fully representative. Changes in growth patterns at interannual to decadal time scales, most likely related to anthropogenic impacts, were observed at B2H (Chimgan, UZ) and KAnw (Kyrgyz Ata, KG) (S1 Fig).

**Fig 5:**
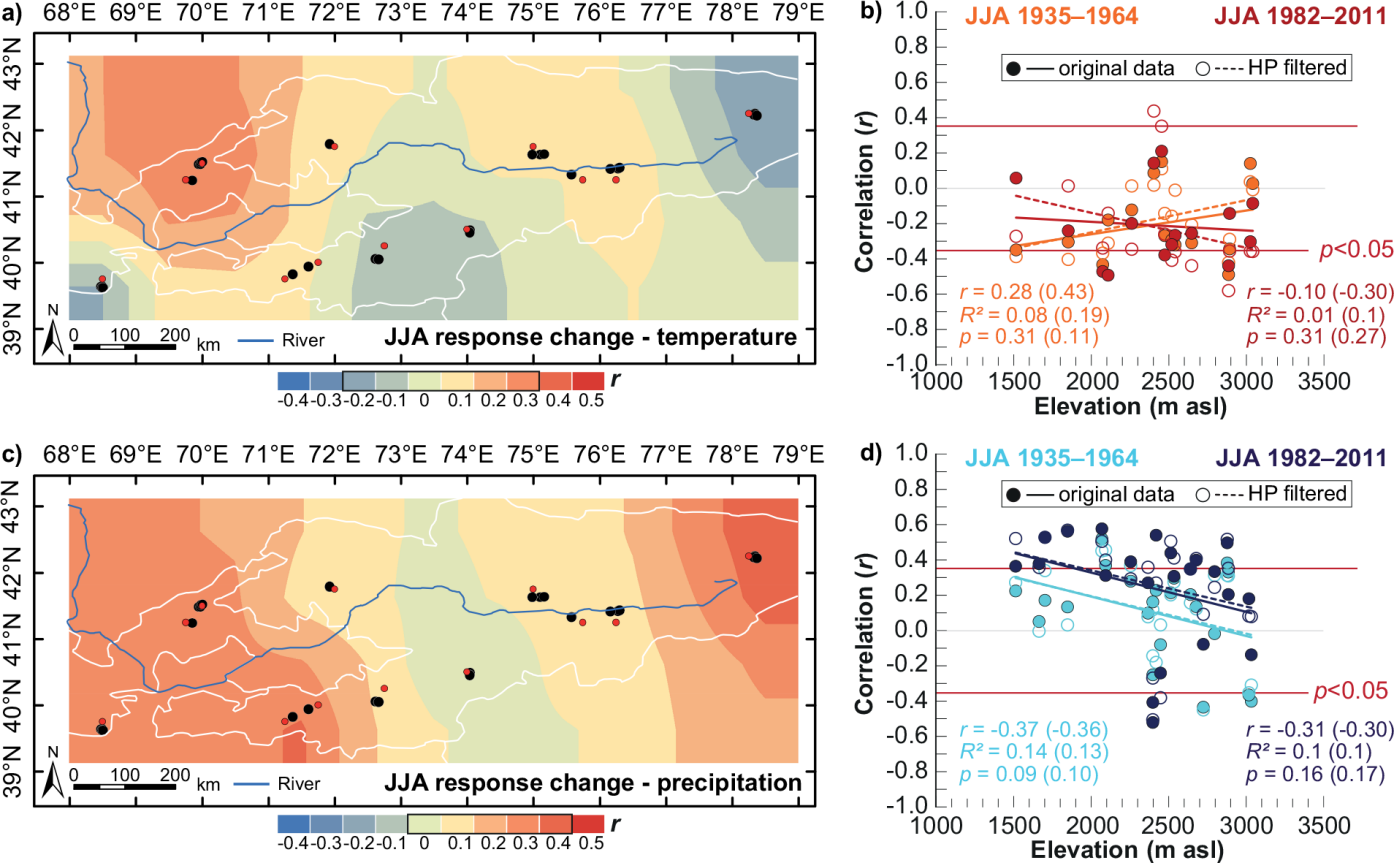
Changes in climate response for the June–August (JJA) season of all 33 sites (black dots) with its closest CRU grid point dataset (red dots) from the 1935–1964 to 1982–2011 period. Differences in **a)** temperature and **c)** precipitation response were spatially interpolated. TRW sites with significant correlations for one of the periods are shown as a function of elevation for **b)** temperature (15 sites) and **d)** precipitation (22 sites) with the unfiltered and filtered (see legend and numbers in brackets) data.

Distinct changes in the climate response of juniper trees were observed across the study area and with respect to elevation (Fig 5). Focusing on the temperature response in JJA, sites located in Zaamin (UZ), Kyrgyz Ata and Karakuldja (southern KG) and Karakol (eastern KG), showed increased negative responses to high temperatures (Fig 5A). In contrast, junipers in Chimgan (UZ) seem to be less stressed by warm temperatures in recent decades, and significant changes in correlations were observed at B2H with *r* = 0.47 (*p*<0.01; from *r* = -0.22 to *r* = 0.25) and B2L with *r* = 0.41 (*p*<0.05; from *r* = -0.35 to *r* = 0.06) (S3A Table). Thus, during 1935–1964, there was an increasingly positive influence of temperatures with increasing altitude (Fig 5B). On the contrary, in the past 30 years at several high elevation sites, junipers have become negatively influenced by high temperatures (Fig 5B), where the most pronounced shifts are seen in the high-pass filtered data (Fig 5B, dashed trend lines).

Regarding the spatial precipitation responses, all regions except Karakuldja (southern KG) showed increased positive correlations in 1982–2011 compared to 1935–1964 (Fig 5C), although this was manifested in different ways. Junipers in the east of our study area, e.g. in Chimgan (UZ), showed increases in the positive correlations with precipitation, i.e. at B1H from *r* = 0.14 to *r* = 0.56 (*p*<0.01) or ML from *r* = 0.17 to *r* = 0.53 (*p*<0.01). However, for junipers in Karakol (eastern KG), where growth was limited by high precipitation during 1935–1964, decreasing precipitation amounts led to improved growing conditions in recent decades. Significant changes in correlation values were found for all three sites but especially for AY (from *r* = -0.36 to *r* = 0.18) and ADJ (from *r* = -0.43 to *r* = -0.08) (S3B Table). Across all altitudes, a tendency towards increasingly positive correlations with precipitation was observed (Fig 5D), suggesting a higher water demand for tree growth also at high elevation sites.

However, the observed changes were not only restricted to summer. Warmer winters had positive influences on juniper growth at seven sites, while increased spring temperatures negatively affected tree growth at four sites (S3 Table).

## Discussion

Our sampling strategy was to include junipers from all age classes within the forest and at stands along different altitudinal gradients, resulting in a new and substantial TRW network for eastern UZ and the whole of KG. At some sites and regions, old and slow-grown trees were found such as at KS (Naryn, KG) or ZNP-JUSE (UZ) with chronologies extending back to AD 1269 and 1482 (un-truncated; S1 Table), respectively. However, further endeavors are necessary to develop well-replicated and robust millennial-long chronologies as shown by Seim *et al* [35]. On the other hand, junipers at some sites were comparatively young in age, for instance at AE and KM (Naryn, KG), pointing to large-scale clear cuts at the end/beginning of the 19^th^/20^th^ century. At both sites, single juniper individuals with ages of ~470 years (AE) and ~350 years (KM) were found. However, strong differences in growth variations between old and young trees hampered successful crossdating, and TRW measurements of those old trees were excluded in this study, restricting our common period to 1935–2011.

It is important to note that the CRU grid points (*n* = 12) located closest to the sampling sites (with an average linear distance of ~ 14 km from the sampling sites) (Fig 1), might not fully represent local climate conditions. By comparing observations from the closest available meteorological stations (average linear distance of ~ 29 km from the sampling sites) in KG and UZ [41] for the baseline period 1961–1990, absolute annual temperatures derived from the CRU grid point data were on average lower (2.3°C) than those obtained from the stations. This is especially evident in winter where the temperature differences were up to 3.3°C, whereas summer temperatures differed only by 1.5°C. For precipitation, overall drier conditions were observed in the CRU data compared to the instrumental data, especially during summer, with an average of 40 mm less rainfall. However, it is also not expected that even meteorological station data might fully represent the climate condition at the sampling sites due to differences in elevation, as discussed by Seim *et al* [35]. Nevertheless, overall long-term trends in temperature and precipitation anomalies for the CRU and station data are in high agreement as shown by Harris *et al* [42].

By choosing two climatological periods at each end of this time window, we detected changes in climate and in the climate sensitivity of the juniper trees. However, less distinct results were obtained when using variations in juniper growth since the trees respond not only to sudden changes in climate but also to human impacts, stand dynamics (i.e. competition) and other abiotic and biotic disturbance factors [45]. Therefore, adding information of tree growth for longer time scales would make the interpretation more robust.

It should be noted that spatial interpolation methods use the maximum range of given values and thus, might be biased by single sites, i.e. data points, while altitudinal differences at the same location are captured only insufficiently. The IDW applied here, however, is a valuable tool to present geographical point data spatially with high efficiency and easy interpretation skills [46].

The observed changes in climate during the past ~80 years are in accordance with previous findings for Central Asia (e.g. [3, 4]). Our results confirm a spatially uniform temperature increase while trends in precipitation regimes are highly diverse across the study region. Shifts in climatic means are additionally enhanced by feedback mechanisms such as increased evaporation due to rising temperatures, changes in snow cover (depth and duration) and in glacier resources [4]. Although increased greenhouse gas emissions caused a warming of the atmosphere over the past century [1], the observed climatic trends and patterns across Central Asia are most likely attributed to 1) feedback mechanisms with the diverse topography and land use [6] and 2) observed changes in atmospheric circulation patterns. In the latter case, our 1982–2011 period concurs with an exceptionally strong positive phase of the North Atlantic Oscillation since the 1980s, which contributed to higher than normal winter temperatures, not only in northern Europe, but also in Central Asia [47]. In addition, an increase in spring to autumn hot extremes was observed in western and central Asia during the last 30 years, associated with an increase in persistent anticyclonic pressure systems [48].

Although changes in climate during the past eight decades were remarkable and comparatively rapid, trends in juniper growth are less obvious and uniform. Combining the obtained results, we can summarize that juniper trees at the sites in Karakol, KG, with humid growing season climate (Fig 1) seem to have benefited from drier summers and consequently, showed increasing growth rates. Conversely, the drier and warmer sites in our study region (i.e. Zaamin, UZ, or Naryn, KG) showed no increases, or slight declines, in annual increments over the analyzed period. Our results, although not being very distinct, are in agreement with general trends observed for semi-arid environments in Asia and Europe. Liu *et al* [49] found significant reductions in tree growth for a compilation of various coniferous species in Inner Asia from 1994 onwards, particularly at semi-arid sites. Also, Galvan *et al* [50], reviewing 66 dendrochronological studies including different broadleaf and coniferous species from more than 640 sites, detected an overall tendency of positive (negative) growth trends at temperate (xeric) sites across the Mediterranean Basin since the 1970’s. Differences in juniper growth at different sites can be linked to different physiological responses regarding gas exchange and water use. Increasing atmospheric CO_2_ concentrations have been related to increased intrinsic water-use efficiency, where increased drought stress can lead to reduced stomatal conductance to compensate water loss at the expense of secondary growth [51–54]. However, detailed studies are needed on 1) all three juniper subspecies as individual tree species seem to perform differently, e.g. [55] and 2) trees from different climate regimes and sites across Central Asia. Moreover, changes in climate responses during winter and spring may additionally modify annual growth as has previously been reported for spruce at tree-line sites in Alaska, where warmer July temperatures lead to a decline in growth and warmer springs enhanced growth [56].

Over the full 1935–2011 period, the growth-climate relationships of the juniper sites generally showed benefiting effects on tree growth from warm temperatures at high altitudes and abundant moisture supply at low elevation sites (Fig 2), agreeing with the general assumption of limiting factors on tree growth [45]. Although the temperature signal in the juniper TRW data was not as strong and statistically significant as, for example, in conifers from tree-line sites in the European Alps [57, 58] or the Tibetan Plateau [59–61], our results are in accordance with findings from earlier studies in KG [28, 62] and UZ [24]. The most likely cause for the relatively low number of statistically significant correlations between juniper growth (i.e. TRW) from high elevations and temperature are temporal instabilities in the climate signal, as indicated by our results. We found distinct response shifts where junipers from high elevation sites were becoming increasingly drought stressed during the past 30 years (Fig 5), a result that not only deviates from the principle of uniformitarianism but also from the principle of limiting factors of tree growth in high mountain environments [45]. Shifting growth-climate responses have also been detected at high elevation conifer sites in Europe (e.g., [63, 64]), restricting the development of temperature reconstructions, or for bristlecone pines near the tree line in the White Mountains of California, USA [65].

Conversely, junipers at lower altitudes showed distinct and persistent drought responses, which has previously been shown for *J*. *seravschanica* in the Zaamin National Park, UZ [35]. Liu *et al* [49] or Liang *et al* [16] noted also an increase in sensitivity to drought and drought-related parameters in Inner Asia. However, increasing temperatures and more frequent droughts that are predicted for the future [1], are likely to increase the risk of forest decline, starting at lower elevations [15]. Moreover, upslope shifts of the juniper forests due to increased tree mortality can be expected, which can occur rapidly and over large areas, as observed for a semi-arid ecotone in northern New Mexico after a severe drought event [66].

The identified growth-climate response shifts in our study, which occurred only within the past 40 to 50 years, most likely indicate impacts of the ongoing climate change on juniper growth in Central Asia. We therefore stress the importance of long-term conservation measures to counteract possible losses or reductions of the mountain juniper forest ecosystems in UZ and KG. Our findings also highlight the need of time-dependency analyses in paleo-climatological studies when using high elevation juniper trees, as well as the integration of growth-climate response shifts when assessing and predicting vegetation dynamics in Central Asia.

## Conclusion

Using a unique and extensive juniper network established for the Tien Shan and northern Pamir-Alay mountain ranges, we detected changes in growth-climate responses during the past eight decades. Junipers at low elevation sites mainly increased their climate sensitivity to drought during summer and the entire growing season. At the highest elevation sites, however, juniper growth was favored by high summer temperatures during 1935–1964, but was limited by increasing drought conditions during the past ~30 years. This change in climate sensitivity of the junipers likely demonstrates the effect of the ongoing climate change, and also explains the low reconstruction skills of high elevation juniper sites in Central Asia. Across the study area, changes in climate response were detected at all regions during summer, but also during winter, spring and autumn. This may be the cause of the spatially non-uniform changes in growth trends across the study region. Finally, our results indicate that a further rise in temperature together with decreasing rainfall amounts, as predicted for the coming decades, will most likely increase the risk of altitudinal range shifts of *Juniperus* spp. and local drought-induced juniper die-backs in this biodiversity hotspot. Consequently, alterations in the floral composition of the temperate coniferous forest biome in Central Asia can be expected. We therefore stress the need to apply adequate long-term measures for ecosystem conservation and to consider this ongoing response shift in paleo-climate and model simulation approaches for this region.

## Supporting Information

S1 Table Sampling sites utilized in this study. Information include country code (CC), latitude (Lat, °N), longitude (Lon, °E), elevation (Elev, m asl), exposition (Exp), species (*Juniperus* sp. (JUSP), *J*. *seravschanica* (JUSE), *J*. *semiglobosa* (JUSM), and *J*. *turkistanica* (JUTU)), the number of trees (*n*) and TRW series (*n*), start and end year over the full and truncated (*n*(*i*) < 5 series) period, interseries correlation (Rbar), mean segment length (MSL), mean sensitivity (MS), Expressed Population Signal (EPS), and highest correlations with climate (*r*_MAX_) for the analyzed 1935–2011 period.
(EPS)

S2 Table **Pearson correlation results for all juniper sites and sites closest grid point data for monthly (current year January to December) and seasonal a) temperature means and b) precipitation sums for the full 1935–2011 period.** Sites within the region are sorted based on increasing longitude and within the region from high to low elevation sites (Kh. = Khaidarkan, S.C. = Sari Chelek, K.A. = Kyrgyz Ata). Seasons include previous year December to current year February (pDJF), March–May (MAM), June–July (JJ), June–August (JJA), September–November (SON), May–September (M–S), April–October (A–O), and the entire year.
(EPS)

S3 Table **Differences in climate response from 1935–1964 to 1982–2011 of all juniper sites for monthly (current year January to December) and seasonal a) temperature means and b) precipitation sums.** Sites within the region are sorted based on increasing longitude and within the region from high to low elevation (Kh. = Khaidarkan, S.C. = Sari Chelek, K.A. = Kyrgyz Ata). Seasons include previous year December to current year February (pDJF), March–May (MAM), June–July (JJ), June–August (JJA), September–November (SON), May–September (M–S), April–October (A–O), and the entire year.
(EPS)

S1 Fig Detrended tree-ring width (TRW) chronologies developed for Uzbekistan (UZ) and Kyrgyzstan (KG), shown for the 20^th^ century. See Fig 1 for specific location of the sites and regions. Grey squares highlight the 1935–1964 and 1982–2011 periods used in the analyses.
(EPS)

S1 File Detrended tree-ring width chronologies used in this study. (XLSX)
